# Genome Analysis of *Klebsiella pneumoniae* Reveals International High-Risk Pandemic MDR Clones Emerging in Tertiary Healthcare Settings in Uganda

**DOI:** 10.3390/pathogens12111334

**Published:** 2023-11-09

**Authors:** Denis K. Byarugaba, Bernard Erima, Godfrey Wokorach, Stephen Alafi, Hannah Kibuuka, Edison Mworozi, Florence Najjuka, James Kiyengo, Ambrose K. Musinguzi, Fred Wabwire-Mangen

**Affiliations:** 1 Makerere University Walter Reed Project, Kampala P.O. Box 16524, Uganda; berima@muwrp.org (B.E.); wokosiki@gmail.com (G.W.); salafi@muwrp.org (S.A.); hkibuuka@muwrp.org (H.K.); emworozi@gmail.com (E.M.); fwabwire@musph.ac.ug (F.W.-M.); 2 College of Veterinary Medicine, Makerere University, Kampala P.O. Box 7062, Uganda; 3 Multifunctional Research Laboratories, Gulu University, Gulu P.O. Box 166, Uganda; 4 College of Health Sciences, Makerere University, Kampala P.O. Box 7062, Uganda; najjukafc@gmail.com; 5 Uganda Peoples’ Defence Forces, Ministry of Defence, Kampala P.O. Box 3798, Uganda; kiyengo2000@yahoo.com (J.K.); akmusinguzi@hotmail.com (A.K.M.)

**Keywords:** multidrug resistant, hypervirulence, virulence, resistance genes

## Abstract

*Klebsiella pneumoniae* is a threat to public health due to its continued evolution. In this study, we investigated the evolution, convergence, and transmission of hypervirulent and multi-drug resistant (MDR) clones of *K. pneumoniae* within healthcare facilities in Uganda. There was high resistance to piperacillin (90.91%), cefuroxime (86.96%), ceftazidime (84.62%), cefotaxime (84.00%), amoxicillin/clavulanate (75%), nalidixic acid (73.68%), and nitrofurantoin (71.43%) antibiotics among *K. pneumoniae* isolates. The isolates were genetically diverse, consisting of 20 different sequence types (STs) and 34 K-serotype groups. Chromosomal *fosA* (for fosfomycin) and *oqxAB* efflux pump genes were detected in all isolates. Two carbapenem resistance genes, *blaNDM-5* and *blaOXA-181* plus extended-spectrum beta-lactamase (*bla_CTX-M-15_*) gene (68.12%), quinolone-resistant genes *qnrS1* (28.99%), *qnrB1* (13.04%), and *qnrB6* (13.04%) and others were found. All, except three of the isolates, harbored plasmids. While the isolates carried a repertoire of virulence genes, only two isolates carried hypervirulent genes demonstrating a low prevalence (2.90%) of hypervirulent strains. Our study demonstrated genetically diverse populations of *K. pneumoniae*, low levels of carbapenem resistance among the isolates, and no convergence of MDR and hypervirulence. Emerging high-risk international pandemic clones (ST11, ST14, ST147, ST 86 and ST307) were detected in these healthcare settings which are difficult to treat.

## 1. Introduction

*K*. *pneumoniae* is continuously evolving, acquiring more resistance and virulence genes as well as other fitness attributes to improve their survival in harsh environments, including the constant onslaught of antimicrobials used against them and the immunological assault. In doing so, this bacterium has evolved into multidrug-resistant (MDR) and hypervirulent clones that are a serious global public health threat. Because of the acquired resistance, many of them have become unresponsive to last-line antibiotic treatment options [1]. This has been exacerbated by the continuous evolution of this pathogen with the acquisition of more resistance and virulence attributes. *Klebsiella pneumoniae* is one of the bacteria that has been listed by the World Health Organization (WHO) as a priority pathogen for research and development for new antibiotics because they have become resistant to multiple antibiotics [2]. It is currently categorized into two pathotypes, namely classical *K. pneumoniae* (cKp) and hypervirulent *K. pneumoniae* (hvKp). These pathotypes are known to differ and the genomic differences have been defined to enable their identification and characterization [3].

The cKp is often associated with hospital outbreaks such as bacteremia, urinary tract infections, and pneumonia, while the hvKp has been reported more in community acquired infections, mainly associated with abscesses such as pyogenic liver abscess, lung abscess, but also endophthalmitis in otherwise healthy individuals [4]. cKP strains are known to undergo frequent genetic changes acquiring multiple resistance genes against several classes of antibiotics including last-resort antibiotics such as carbapenems. Some of these have been classified as high-risk clones of international importance such as the CC258 clone which comprises several sequence types.

The hvKP have been mainly associated with community acquired infections and are highly invasive with a hypermucoviscous phenotype [4]. However, these have increasingly been reported in healthcare settings and have spread globally, in addition to acquiring multiple resistance genes that have further complicated treatment outcomes [5]. They carry several virulence factors including siderophores (enterobactin, yersiniabactin, salmochelin); rmpA conferring mucoid phenotype, aerobactin, allantoin metabolism, a fimbrial gene cluster, conjugative element (*ICE*/*ICEKp1*), and *kfu* operon, most of which are carried on virulence plasmids [6]. Complex cases caused by hvKP have been reported with liver abscesses, others with extrahepatic complications such as pyogenic ophthalmia leading to blindness, prostate abscesses, purulent meningitis, and necrotizing fasciitis [7]. 

The evolution and global spread of these hypervirulent (hv) and MDR clones is a major concern. Previously the hypervirulent clones evolved independently from the MDR strains [8]. Convergence of the MDR and hypervirulent clones is increasingly being reported globally [9]. This is said to be through the acquisition of both hypervirulent and resistance determinants on mobile genetic elements, especially plasmids with hvKp acquiring resistance to multiple classes of antibiotics and cKp acquiring several virulence genes [10]. This evolution of *K. pneumoniae* has resulted in several high-risk clones separated into well-defined (i) multidrug-resistant clones such as the ST258, ST147, ST101, and (ii) hypervirulent clones such as ST23, ST65, ST86, with corresponding clonal groups: CG258, CG147, and CG101 for the MDR-KP and CG23, CG65, and CG86 for the hv*Kp* [9]. Additional high-risk clones are increasingly being reported in different parts of the world, such as ST307, ST25, ST20, and ST37 with frequent outbreaks where infection and prevention/control measures are not maximal in humans but also in synanthropic animals in Africa [11,12]. We have recently demonstrated K1 and K2 serotypes in Uganda which are commonly associated with MDR strains with a high proportion (23%) being resistant to carbapenems [13]. Most hvKp belong to clonal complex 23 (CC23) of the K1 serotype. They are said to have evolved from a single common ancestor and spread globally through multiple international transmissions. These are distinct from K2 hvKp that have been demonstrated to be encoded on a large virulence plasmid with two siderophores, aerobactin, salmochelin, and *RmpA* genes [14]. The convergence of the high virulence and MDR which makes them more fit for survival has been said to be driven by the exchange of the plasmids in both directions. MDR strains acquiring hvKp virulence genes or hvKp strains acquiring MDR resistance genes constitute several high-risk clones [15]. The current study sought to establish the genomic structure of the *K. pneumoniae* isolated from patients seeking healthcare services in tertiary healthcare facilities in Uganda, tracing the evolution and potential convergence of the hvKp and MDR clones and transmission of these within the healthcare facilities.

## 2. Materials and Methods

### 2.1. Study Sites

The isolates used in this study were recovered under the antimicrobial surveillance program of Makerere University Walter Reed Project, which has been running since 2012. The samples were collected from patients who visited one of four government hospitals that offer healthcare services to everyone. Kiruddu National Referral Hospital is a 200-bed capacity national referral hospital with fourteen outpatient clinics that attend to about 250 patients daily, located in the capital city of Kampala in the central region of Uganda established in 2013. The hospital provides healthcare services to patients from Kampala and other regional referral healthcare facilities. Gulu Regional Referral Hospital is a 370-bed capacity regional hospital taking care of referrals for the districts of Amuru, Gulu, Kitgum, Lamwo, Nwoya, and Pader in northern Uganda. Bombo Hospital, with a 250-bed capacity, is a military hospital in Uganda and primarily caters to the healthcare needs of military personnel, their families, and the communities around Luweero district. Bwera General Hospital is a 200-bed capacity public healthcare facility located in the Western Region of Uganda in the Kasese District on the border with the Democratic Republic of Congo. It provides medical services to patients from the Kasese District as well as those from the neighboring Democratic Republic of Congo.

### 2.2. Bacteria Isolation

Samples were directly inoculated onto MacConkey agar (Oxoid, Remel Inc., 12076 Santa Fe Drive, Lenexa, KS, USA) and incubated at 37 °C for 24 h. A single mucoid colony of lactose-fermenting bacteria were sub-cultured on Eosin methylene blue (EMB) agar (Oxoid, Remel Inc., 12076 Santa Fe Drive, Lenexa, KS, USA) at 37 °C for another 24 h. Large, mucoid, pink-to-purple colonies with no metallic green sheen on EMB agar were picked and inoculated onto API- 20E kits and presumptively identified as *Klebsiella pneumoniae* (bioMérieux, Boston, MA, USA). Pure colonies were stored in brain heart infusion (BHI) broth (Oxoid, Manchester, UK) containing 50% glycerol at −80 °C until further analysis.

### 2.3. Antimicrobial Susceptibility Test

The isolates were subjected to antimicrobial susceptibility testing against 17 antibiotics by disc diffusion assay as previously described [16] on Mueller–Hinton agar (MHA) (Oxoid, Manchester, UK). A suspension of 0.5 McFarland standard turbidity was spread on the surface of MHA plates using a sterile cotton swab. Antibiotic discs Ampicillin (AMP10 µg), Amikacin (AMK30 µg), Amoxicillin-clavulanic acid (AMC20/10 µg), Cefotaxime (CTX30 µg), Ceftazidime (CAZ30 µg), Ceftriaxone (CRO30 µg), Cefuroxime (CXM 30 µg), Ciprofloxacin (CIP5 µg), Chloramphenicol (C30 µg), Gentamicin (C10 µg), Trimethoprim-sulfamethoxazole (SXT1.25/23.75 µg), Tetracycline (TE30 µg), Nalidixic acid (NA30 µg), Nitrofurantoin (F300 µg), Imipenem (IPM10 µg), Ertapenem (ETP10 µg), and Meropenem (MEM10 µg) were placed on the surface of MHA agar plates and incubated at 37 °C for 24 h. The zone of inhibition was measured to the nearest millimeter and interpreted based on the guidelines of the Clinical and Laboratory Standards Institute (CLSI M100 Ed33) [17] together with the *E. coli* ATCC 25922 reference strain.

### 2.4. Selection Criteria

A total of 391 *K. pneumoniae* isolates were recovered during the study period. Of these, 69 (17%) showed resistance to three or more classes of antimicrobial agents and were classified as multidrug resistant (MDR) accordingly [18] and selected for whole genome sequencing for further characterization. The MDR *K. pneumoniae* bacteria were isolated from outpatients, inpatients, patients in wards, patients in the emergency unit, and patients in the intensive care unit (ICU). All *K. pneumoniae* isolated from outpatients were considered as community-acquired sources of infection. Similarly, all *K. pneumoniae* isolated from wards, ICU, and inpatients were considered healthcare-acquired sources of infection.

### 2.5. Library Preparation

The bacterial isolates were sub-cultured on Luria Broth (LB) media for 48 h at 37 °C and the cell masses were harvested in 1.5 mL tubes and proceeded with DNA extraction from the isolates using DNeasy UltraClean microbial kit (Qiagen, Germantown, MD, USA). The concentration of the DNA was measured with the aid of the Kapa library quantification kit Illumina/Bio-Rad iCycler (Roche Diagnostics, Indianapolis, IN, USA) in a CFX96 real-time cycler (Bio-Rad, Hercules, CA, USA). The concentration of DNA was normalized to a uniform concentration. The normalized DNA was used in the preparation of nucleic acid libraries using Kapa HyperPlus library preparation kits (Roche Diagnostics, Indianapolis, IN, USA). The final concentration of the libraries was determined with the Kapa library quantification kit and then normalized to a uniform concentration. The samples were sequenced as pair-end on Illumina NextSeq at Walter Reed Army Institute (WRAIR) Multidrug-Resistant Organism Repository and Surveillance Network (MRSN).

### 2.6. Sequence Quality Check, Assembly, and Taxonomic Assignment

Fastq raw reads were assessed for quality using fastqc tools. Trimming with Btrim was then performed to remove regions with poor base call quality and Illumina sequence adapters [19]. Consensus contigs were created using Newbler (v2.9) assembly, utilizing reads from trimming [20]. The taxonomy of the isolates was inferred from the assembled genome sequences by matching them to similar reference genome sequences within the Genome Taxonomy Database (GTDB). An average nucleotide identity (ANI) of ≥95% between the query sequences and those of their close match in the GTDB indicates they are of the same species.

### 2.7. Genome Sequence Analysis

The population structure of the isolates was established by determination of Multi-locus sequence typing (MLST), K-serotype, and O-serotype grouping using Kleborate and Kaptive [21,22]. The clonal groups (CGs) were determined based on the scgMLST629_S classification scheme of the Institut Pasteur [23]. The acquired antibiotic-resistant genes within the isolates were determined using both ResFinder 4.1 and Kleborate [22,24]. The resistance score was determined for each isolate https://github.com/klebgenomics/Kleborate/wiki/Scores-and-counts (accessed on 2 June 2023). A score of zero was assigned when the isolate carried no ESBL and carbapenemase-resistant gene regardless of colistin resistance. A score of one was when ESBL gene was present but with no carbapenemase (regardless of colistin resistance). A score of two was given when carbapenemase was present without colistin resistance (regardless of ESBL genes or OmpK mutations). A score of three was given when carbapenemase and colistin resistance genes were present (regardless of ESBL genes or OmpK mutations). We utilized mlplasmids v2.1.0 to pinpoint the origin of contigs, either from plasmids or chromosomes, and further confirmed via NCBI blast [25]. Additionally, we examined both the plasmid contigs and chromosomes for AMR genes to precisely identify the location of resistance genes. The different contigs were annotated with DFAST v1.2.18 standalone to identify the location and arrangement of AMR genes [26]. The organization of AMR genes within contigs was mapped and visually represented using the CGView within Proksee https://proksee.ca/ (accessed on 20 June 2023) [27,28]. Kleborate and VirulenceFinder v2.0 databases were both employed to detect the existence of diverse virulence genes in the genome of *K. pneumoniae* isolates [29]. Virulence score was assigned to the isolate based on the presence or absence of yersiniabactin (*ybt*), colibactin (*clb*), and aerobactin (*iuc*) as described in Kleborate https://github.com/klebgenomics/Kleborate/wiki/Scores-and-counts (accessed on 2 June 2023). A score of zero was assigned when the isolate lacked any of the yersiniabactin, colibactin, and aerobactin genes. A score of one was assigned when only yersiniabactin gene was present. A score of two was assigned when yersiniabactin and colibactin genes were present. A score of three was assigned when aerobactin was present without yersiniabactin and colibactin. A score of four was given when both aerobactin and yersiniabactin were present without colibactin. A score of five was assigned when both yersiniabactin, colibactin, and aerobactin were present. Using roary v3.11.2, the pangenome and core genome of the *Klebsiella pneumoniae* isolates were determined [30]. The Reference sequence Alignment-based Phylogeny builder (REALPHY v1.13) was used to build phylogenetic trees [31]. First REALPHY v1.13 maps the sequences against the reference via bowtie2 and generates multiple alignments from which a phylogenetic tree was inferred via PhyML. The phylogenetic tree was imported into Interactive Tree of Life https://itol.embl.de/ (accessed on 12 October 2023) for viewing and annotation.

### 2.8. Statistical Analysis

Fisher’s exact test was used to determine differences in the occurrence of antimicrobial resistance genes in the four hospitals. The Kruskal–Wallis test was used to compare the number of resistance gene classes, number of resistance genes, and relative abundance of resistance gene classes of *Klebsiella pneumoniae* isolated from different hospitals. The difference in the number of resistant classes with virulence scores and the number of resistant genes with virulence scores was determined with the Kruskal–Wallis test. All statistical analyses were performed using R 4.0.3 [32].

## 3. Results

### 3.1. Characteristics of the MDR Isolates Used in the Study

Out of the total n = 69 MDR *K. pneumoniae* isolates, n = 39 were from females while n = 30 were from males. Most of these *K. pneumoniae isolates* (n = 28) were from patients at General Military Hospital, followed by Kiruddu National Referral Hospital (n = 21) and Bwera General Hospital (n = 18). Only n = 2 isolates were from patients at Gulu Regional Referral Hospital. The majority of MDR *K. pneumoniae* were isolated from outpatients (n = 20) and from inpatients (n = 36), respectively. Only a few MDR *K. pneumoniae* were isolated from patients in wards (n = 3), emergency unit (n = 1), clinic (n = 1), and ICU (n = 1). The majority of *K. pneumoniae* were isolated from pus (n = 30) and urine (n = 27), while a small number of isolates were recovered from sputum (n = 2), wound (n = 2), blood (n = 2), high vaginal swabs (n = 2), endocervical swabs (n = 3), and medical device (n = 1) (Table S1).

### 3.2. Antibiotic Susceptibility of the Isolates

The results of the antibiotic resistance testing indicated that all isolates (100%) were resistant to aztreonam. The following drugs also showed high levels of resistance: cefuroxime (86.96%), piperacillin (90.91%), ceftazidime (84.62%), and cefotaxime (84.00%). Similarly, resistance to amoxicillin/clavulanate (75.00%), nalidixic acid (73.68%), and nitrofurantoin (71.43%) were also more prevalent. Resistance to chloramphenicol (65.38%), gentamicin (57.89%), and amikacin (40%) showed a moderate amount of resistance while resistance to carbapenems imipenem (17.86%) and meropenem (25.0%) were low (Table S2).

### 3.3. Klebsiella Sequence Types (STs), K-Serotype, and O-Serotype

**Figure 1:**
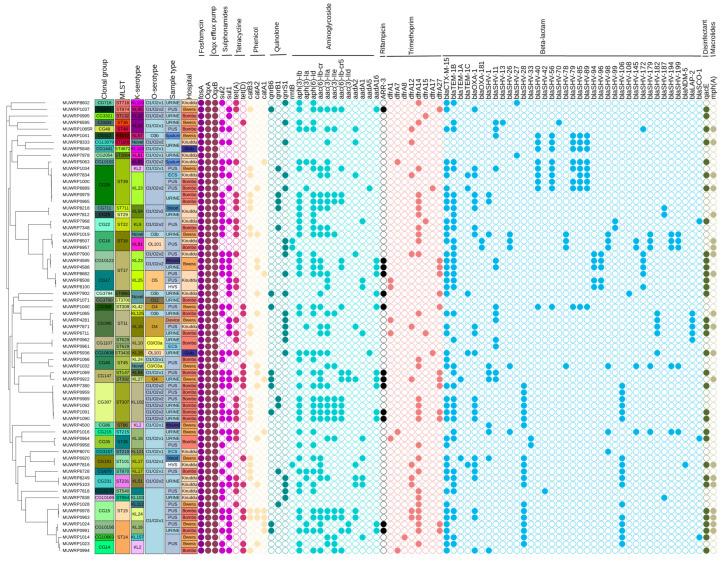
SNP-based phylogenetic tree from core genes of 69 *Klebsiella pneumoniae* isolates analyzed in this study characterized by clonal group, MLST, serotype, sample type, healthcare care facility and antimicrobial resistance genes (absent, open circle; present, filled colored circle according to the class of AMR genes).

The isolates displayed a high level of diversity, with 20 different sequence types (STs) identified. Several ST groups were identified in the isolates, among which high-risk sequence types ST307, ST86, and ST147 were identified in addition to the others such as ST16, ST215, ST309, ST3700, ST392, ST412, ST48, and ST874 (Figure 1). A total of 34 K-serotype groups that define the *K. pneumoniae* population were found. These groups are predominantly associated with the K loci identified in the K1-K77 capsule serotype reference strains. In the study, the capsules observed were mainly KL16 (n = 5), KL23 (n = 7), KL15 (n = 4), KL24 (n = 3), KL25 (n = 3), and KL81 (n = 3) capsules in addition to the hypervirulent-associated capsule type KL2 (n = 4). A small percentage of isolates were among K serotypes where the serological capsule groups are still unidentified. In this category, the K102 is the most frequent with a total of five occurrences, while KL112, KL125, and KL157 each have one or two occurrences within the serotype group. Out of the eight identified O-serotype groups, a significant proportion were O1/O2v1 (n = 26) and O1/O2v2 (n = 24). The remaining serotypes, including O3/O3a (n = 3), O3b (n = 4), O4 (n = 5), O5 (n = 3), and OL101 (n = 3), were found at low frequencies among the population of *K. pneumoniae* isolates (Figure 1).

### 3.4. Clonal Groups

We identified up to four high-risk clonal groups and among them were CG307, CG86, CG147, and CG101. None of our isolates belonged to the common MDR CG258. The CG307 was the most common (8.69%; n = 6) of the isolates and were all from patients at General Military Hospital Bombo; all these isolates had capsular types (KL102 and O1/O2v2) isolated from pus and urine samples only. The two CG147 were found in two isolates, including ST392 and ST147, which were also recovered from urine and pus samples (Figure 1). There were two isolates recovered that belonged to CG101 clonal group from blood and vaginal swabs obtained from inpatients at Bwera General Hospital and Kiruddu National Referral Hospital, respectively, and carried capsular types K17 and O1/O2v1 (Figure 1). One of the hypervirulent strains was classified as a high-risk CG86, while the other was linked to CG412. Similarly, the CG39 clonal group with KL23 and O1/O2v2 capsular types was observed in 8.69% of the isolates. Most patients with CG39 were from General Military Hospital Bombo; however, there were a few isolated instances at Kiruddu National Referral Hospital and Bwera General Hospital. The detected CG39 instances were mostly discovered in pus and urine, although they were also isolated from endocervical swabs. Five percent (5%) of the isolates belonged to the CG340 and were related to the ST11 sequence type. Except for one isolate that contains K125 and O3b capsular types, the CG340 mostly carried K15 and O4 capsular types. The CG340 clonal group was isolated from several sample types, including urine, pus, and medical devices, and was shown to be dispersed in all the hospitals. Within a single sub-lineage (SL14), there were two isolates that were part of the CG14 clonal group. The KL2-associated capsular type is present in the CG14 clonal group. CG14 isolates were collected from General Military Hospital Bombo and Bwera General Hospital. Three isolates, all recovered from pus samples, belonged to the CG15 group, which also corresponded to sub-lineage SL15. One CG15 isolate from Bwera General Hospital contained the KL112 capsule type, whereas two CG15 isolates from General Military Hospital Bombo all carried the KL24 capsule type. Three isolates associated with ST16 are members of the CG16 clonal group. A urine-derived CG16 isolate from a patient at Bwera General Hospital carried capsule type KL51 and O3b, in contrast to two of the CG16 isolates derived from pus samples taken from a patient at General Military Hospital Bombo and Kiruddu National Referral Hospital that contained capsular type KL81 and OL101. All the CG17 isolates (n = 3) came from Kiruddu National Referral Hospital and had uniform capsular type KL25 and O5 regardless of the sample types which were pus (n = 2) and vaginal swab (n = 1) from which they were derived.

### 3.5. Detection of AMR Genes

**Figure 2:**
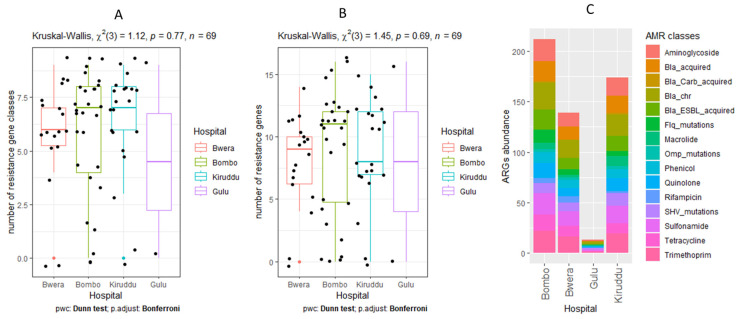
Comparison of the number of resistance gene classes (**A**), number of resistance genes (**B**), and relative abundance of resistance gene classes (**C**) of *Klebsiella pneumoniae* isolated from the different hospitals. Bwera: Bwera General Hospital, Bombo: General Military Hospital Bombo, Gulu: Gulu Regional Referral Hospital, and Kiruddu: Kiruddu National Referral Hospital.

Isolates were examined for resistance genes and their distribution. The number of acquired antibiotic resistant gene classes has a median value of 7 (mean of 5.96) and the number of resistance genes has a median of 10 (mean of 8.55) and did not significantly vary in regard to hospitals (Figure 2). Antibiotic resistance gene classes including fosfomycin, sulphonamides, beta-lactams, aminoglycosides, quinolones, tetracyclines, macrolides, phenicol, trimethoprim, and sulphonamides were found to be present. We detected two genes that confer resistance to carbapenemase in three of the sixty-nine isolates sequenced: *blaNDM-5* (n = 1) and *blaOXA-181* (n = 2), while the extended-spectrum beta-lactamase gene (*blaCTX-M-15*) was a very common resistant gene detected in 68.12% of isolates.

As is typical, all of the *K. pneumoniae* isolates contained the *fosA* gene as well as the *OqxA* and *OqxB* genes. The *sul1* (52.17%) and *sul2* (59.42%) genes, known to confer resistance to sulphonamides, were found to be randomly distributed among the O and K serotypes, with the exception of the O3/O3a, OL101, and O3b serotypes, which exhibited a restricted distribution of these genes. The *tet(A)* gene (39.13%) and the *tet(D)* gene (18.84%) were the exclusive tetracycline-resistant genes present. *Tet(D)* was mainly found in isolates from Bwera General Hospital and General Military Hospital Bombo. The occurrence of chloramphenicol-resistant genes *catB3* (31.88%), *catA2* (20.29%), and *catA1* (11.59%) was random among *K. pneumoniae* isolates and had no pattern of association with hospitals and sample types (Figure 1). The three most prevalent quinolone-resistant genes identified were *qnrS1* (28.99%), *qnrB1* (13.04%), and *qnrB6* gene (13.04%). In comparison to General Military Hospital Bombo and Bwera General Hospitals, isolates from Kiruddu National Referral Hospital had a considerably (*p* = 0.00) higher prevalence of the *qnrS1* gene. The isolates exhibited multiple aminoglycoside-resistant genes with the highest prevalence rates being *aph (3)-Ib* (55.07%), *aph (6)-Id* (53.62%), and *aac(6)-Ib-cr* (46.37%). The *aac(3)-IIa*, *aac(3)-IIe*, and *aac(6)-Ib-cr5* genes were detected at a uniform frequency of 24.64%. The *aadA16* (18.84%), *aadA2* (15.94%), *aadA1* (13.04%), and *aph(3)-Ia* (13.04%) were randomly detected among the isolates. Aminoglycoside gene *aac (3)-IId* marginally occurs at a frequency of (5.80%). The *ARR-3* gene for rifampicin resistance occurred among 18.84% of isolates and was randomly distributed within the different O-serotypes except for O3/O3a and OL101. Trimethoprim-resistant genes were detected as follows: *dfrA14* (42.03%), *dfrA27* (18.84%), *dfrA12* (15.94%), *dfrA1* (7.25%), and *dfrA7* (4.37%). Also, various *blaSHV* gene alleles were found distributed randomly among the isolates (Figure 1).

### 3.6. Mutations Associated with Antibiotic Resistance

Mutations in the *GyrA* and *ParC* genes, which confer resistance to fluoroquinolone antibiotics, were detected in a number of isolates. A total of 26.96% of the isolates were detected with the *GyrA* mutation. Hospitals determine the level of occurrence of *GyrA* mutation. For example, only two samples from General Military Hospital Bombo lacked the *GyrA* mutation, but the vast majority did. The *GyrA* mutation was also found in Kiruddu National Referral Hospital and Bwera General Hospital but only at a low level. *ParC* mutations were found in 23 isolates in total, and the pattern of occurrence followed that observed with *GyrA* mutation with respect to hospitals. The majority of isolates derived from General Military Hospital Bombo were detected to carry *parC* mutation compared to samples from Kiruddu National Referral Hospital and Bwera General Hospital. Beta-lactamase-resistant *OmpK35* and *OmpK36* mutations were found in eight and two isolates, respectively, among different sequence types (ST16, ST14, ST874, ST11, ST307, ST4961).

### 3.7. Distribution of Plasmids and Other Mobile Genetic Elements

**Figure 3:**
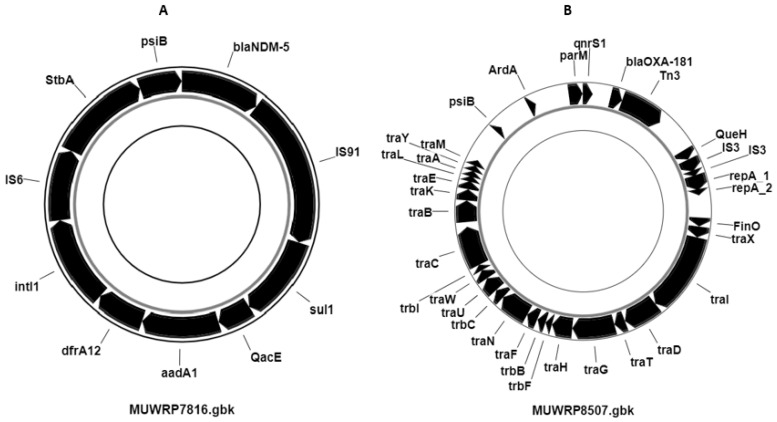
Partial annotation of the plasmid from isolates MUWRP7816 (**A**) and MUWRP8507 (**B**) carrying carbapenemase-resistant gene *blaNDM-5* and *blaOXA-181,* respectively, with other acquired antimicrobial-resistant genes (*aadA1*, *sul1*, *QacE*, and *dfrA12*) and quinolone-resistant (*qnrS1*).

While *fosA, oqxA,* and *oqxAB* resistance genes were chromosomally encoded and present in all isolates, all but three isolates carried plasmids (95.65%) and several antibiotic resistance genes mobilized within the plasmids. Among those were genes for extended-spectrum β-lactamases (*ESBL*) and carbapenemase-resistant genes. A 37,151 bp plasmid was identified to carry the carbapenemase resistance gene (*blaNDM-5*) on strain MUWRP7816 that resembled *p_dm485b_NDM5* plasmid (100% identity). Additionally, the plasmid containing the *blaNDM-5* gene also harbored three other AMR genes (*aadA2*, *sul1*, and *dfrA12*) (Figure 3). The presence of the *blaOXA-181* gene, resistant to carbapenemase and also located on a plasmid, was detected in two isolates. Strain MUWRP8507 carried a 96,594 bp plasmid that contained the *blaOXA-181* gene. This plasmid had 99% identity with plasmid *pKPX-2* (accession: AP012056). Similarly, the carbapenemase-resistant gene-carrying plasmid in MUWRP6957 was 12,708 bp, matched with plasmid *pBC947-OXA-181* (accession: MK412920) with 99.99% identity. The two plasmids carrying the carbapenemase gene *blaOXA-181* were also found to be linked with the *qnrS1* gene (Figure 3).

### 3.8. Characteristics and Distribution of Virulent Genes

**Figure 4:**
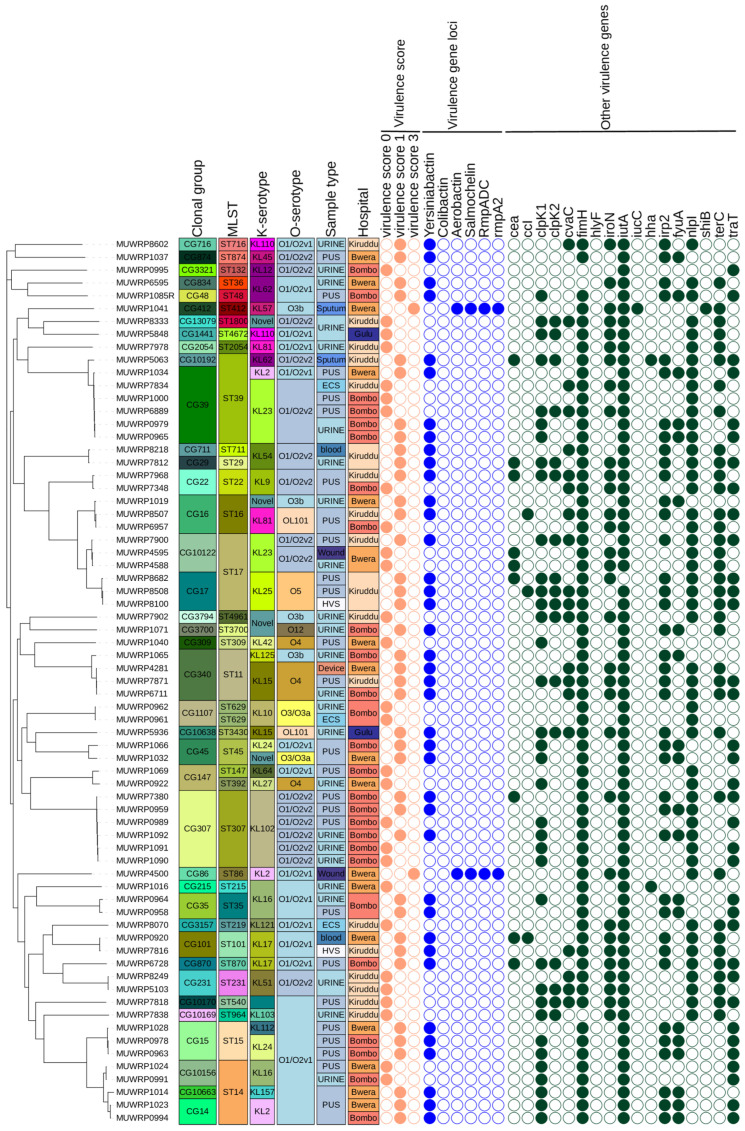
SNP-based phylogenetic tree from core genes of *Klebsiella pneumoniae* isolates analyzed comparing isolates for their virulence score, virulence genes, sequence type, serotypes, and sample isolation source (absent, open circle; present, filled colored circle according to the class of AMR genes).

In addition to the spectrum of virulence genes, five genetic markers associated with more severe infections (known as “hypervirulent” traits) were specifically analyzed. Our findings showed that only two out of sixty-nine isolates (2.90%) carried three hypervirulent genes: Aerobactin (*iuc1*), Salmochelin (*iro1*), and hypermucoidy (*RmpADC* and *rmpA2*). The two isolates that harbored the *iuc1*, *iro1*, *RmpADC*, and *rmpA2* gene loci in their genome had a virulence score of three and they belonged to ST412 and ST86 groups. The hypervirulent strains were isolated from patients receiving healthcare services from Bwera General Hospital. One strain was community-acquired (MUWRP1041), while the other was healthcare-associated (MUWRP4500). The yersiniabactin (*ybt*) gene loci, which produce siderophores, were found in over half (57.97%) of the *Klebsiella pneumoniae* population. Many of the isolates displayed a virulence score of one, largely due to the existence of the yersiniabactin gene (Figure 4). These loci were randomly distributed among different sequence types and serotypes. Eight distinct yersiniabactin siderophore gene loci (*ybt10*, *ybt13*, *ybt14*, *ybt16*, *ybt26*, *ybt5*, *ybt8* and *ybt9*) were mobilized into different *ICEKp* variants. The *ybt9* (n = 14) gene site, localized on *ICEKp3*, and identified in a total of 10 distinct STs, was the dominant yersiniabactin gene site. *Ybt10* was carried on *ICEKp4* and was mainly detected among isolates with sequence types (ST307, ST15, ST45, ST29 and ST16). One isolate with locus ybt10 (ST16) had a resistance score of two while the majority had a score of one or zero. A total of n = 7 isolates with the *ybt14* gene carried mainly on *ICEKp5*, except for a single strain (ST874) was detected with *ICEKp12*. The *ybt14* gene position was detected in ST17, ST39, ST14, and ST3430. *Ybt16* was associated with *ICEKp12* and was detected in six isolates (ST17, ST716, ST307, ST39 and ST14). Meanwhile, *ybt26* (*ICEKp18*), *ybt13* (*ICEKp2*), and *ybt8* (*ICEKp9*) were detected in one isolate, respectively.

**Figure 5:**
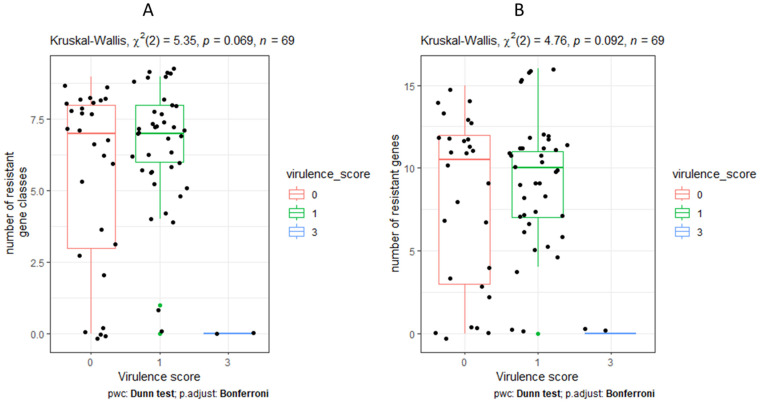
Comparison of number of resistant classes with virulence score (**A**) and number of resistant gene with virulence score (**B**).

We also looked for other genes associated with virulence besides Aerobactin (*iuc1*), Salmochelin (*iro1*), and hypermucoidy (*RmpADC* and *rmpA2*) and observed that ferric aerobactin receptor (*iutA*) gene occurred in 98.55% and type 1 fimbriae (*fimH*) gene occur in 95.65% of isolates. A total of 71.01% of isolates had the lipoprotein precursor gene (*nlpl*). The *terC* gene for Tellurium ion resistance protein and *ClpK1* gene for heat shock survival occur in 52.17% of the isolates, respectively. A total of 50.72% of isolates had the *traT* gene for complement resistance to outer membrane protein. Only two isolates had hemolysin expression modulator (*Hha*/*rmoA*) gene, one of which was from Kiruddu National Referral Hospital and Bwera General Hospital with the *Hha*/*rmoA* gene and KL16/O1/O2v1 serotype. The only isolate with *Shigella flexneri SHI-2* pathogenicity island (*shiB*) genes was isolated from sputum sample, and it belonged to ST412 and serotype (KL57/O3b). In isolates with a virulence score of three, acquired antimicrobial resistance genes were limited. However, a variety of acquired antibiotic-resistant gene classes were present in isolates with virulence score ratings of zero and one. For isolates with a virulence score of zero and one, the median AMR gene classes and AMR gene count were 7.0 and 10.5, respectively (Figure 5). However, the observed difference was not significant and only highlighted the disparity in the occurrence of acquired antibiotic-resistant genes with virulence scores.

### 3.9. Phylogenetic Relationships

**Figure 6:**
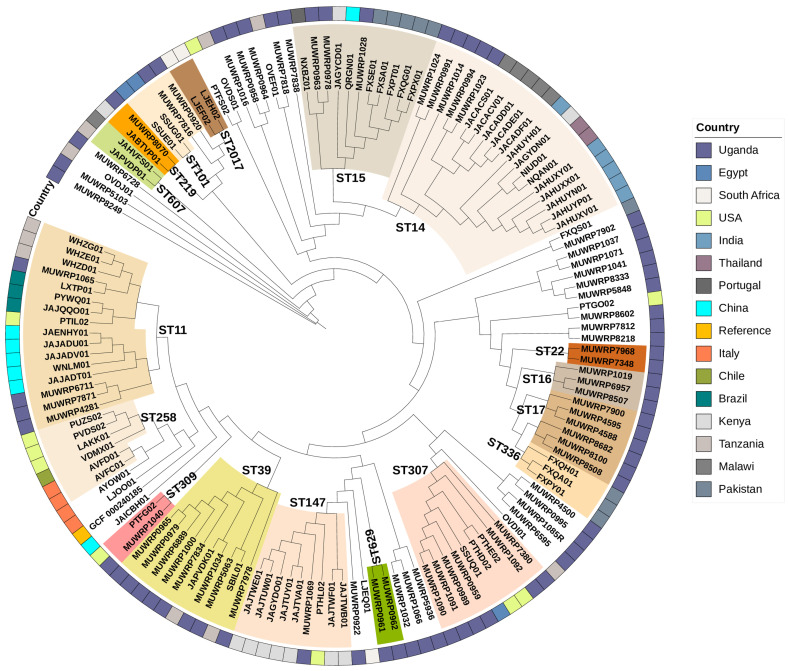
Phylogenetic relationship of *Klebsiella pneumoniae* isolated from Uganda in a global context demonstrating the diversity of isolates. The Reference sequence Alignment-based Phylogeny builder was used to map the sequences against the reference via bowtie2. Multiple alignments of core genes were generated from which a phylogenetic tree was inferred via PhyML.

The phylogenetic relationship of the 69 Ugandan isolates was compared with contemporary isolates with sequences available in the GenBank from elsewhere in the world. The various STs grouping observed in Uganda closely grouped with strains reported elsewhere. ST11 cluster was dominant with isolates from different countries including Uganda, China, Brazil, and USA (Figure 6). Similarly, the ST14 cluster had isolates from Thailand, India, Kenya, Malawi, and Uganda. The Tanzanian isolates (SBIL01 and JAPVDK01) group together with eight isolates from Uganda in the ST39 cluster (Figure 6). The ST307 cluster contains strains from Uganda, Egypt, and the USA. The ST15 cluster contains isolates from Uganda, Kenya, and Pakistan.

## 4. Discussion

While considered opportunistic pathogens, *Klebsiella pneumoniae* causes several and sometimes severe healthcare-associated and community-acquired infections including bloodstream infections, pneumonia, and urinary tract infections. In this study, we characterized MDR *Klebsiella pneumoniae* isolates at whole genome level and established their genetic diversity, resistome, virulome, and population structure to identify the extent of the threat of the international pandemic/epidemic high-risk clones with possible convergence of resistance and hypervirulence that is complicating management of these infections. We confirmed the high genetic diversity of *K. pneumoniae* isolated from this study with a broad number of sequence types, as diverse as the clonal groups as well as the serotypes. The evolution and diversity of *K. pneumoniae* has been reported in many parts of the world, including Africa [1,12,15,33]. Some of these studies have identified international high-risk clones such as CG258 and CG14 as well as the ST307, ST11, ST14, ST15, ST307, ST231 and ST147 [34]. Many of these are characterized by high clonal diversity, antimicrobial resistance, virulence, and/or hypervirulent factors [1]. In this study, we identified high-risk clones of ST11, ST14, ST147, and ST307, with ST307 being the predominant. ST307 was first reported in late 2000 in the Netherlands (in 2008) and later on in several parts of the world, including Africa. It has since been reported to be endemic in many parts of the world including Italy, Colombia, the United States, and South Africa, while ST147 is endemic in India, Italy, Greece, and certain North African countries; both continue to spread elsewhere [35,36]. The finding of these high-risk clones in Uganda is not surprising given the global dissemination of these clones and the ease with which they spread and transmit within healthcare facilities but also in communities. They pose a serious threat to the management of infectious diseases. Most of them are multidrug resistant and have been identified by the World Health Organization to be of critical priority due to their resistance to carbapenems and third-generation cephalosporins with limited options for treatment [37].

Indeed, most of our isolates were resistant to multiple antibiotics and carried multiple resistance genes. The majority were ESBL-resistant, characterized by the carriage of the *bla_CTXM-15_*. In addition, they carried many antibiotic resistance genes, which phenomenon is widely acknowledged with more than 100 different acquired AMR genes identified in *K. pneumoniae* mostly carried by AMR plasmids [1]. Efflux pump encoded resistance mechanisms are widely found among many bacterial species playing a role in both intrinsic and acquired resistance to antimicrobials by lowering intracellular antibiotic concentrations. Since 2004, when *OqxA/B* efflux pumps were first reported to be plasmid-encoded, multidrug efflux pumps have been reported regularly across the globe conferring resistance to multiple drugs, detergents, and disinfectants including quinoxalines, quinolones, tigecycline, nitrofurantoin and chloramphenicol [38]. All the isolates in our study carried *fosA* and *oqxAB* genes for fosfomycin and efflux pump genes, respectively, that were all chromosomally encoded. While it has been suggested that efflux pumps are an important mechanism of fosfomycin resistance in other bacteria such as *A. baumannii*, it is generally understood these genes are intrinsic to all *Klebsiella* species and may not confer fosfomycin/fluoroquinolone resistance in these species. Fosfomycin is a broad-spectrum bactericidal antibiotic that inhibits cell wall synthesis, and its resistance is said to occur due to mutation in the drug uptake system or by the acquisition of fosfomycin-modifying enzymes.

Besides the widespread occurrence of *ESBL* strains characterized by the carriage of *bla_CTXM-15,_* we detected only three isolates carrying carbapenemase genes; specifically, the *blaNDM-5* (one MUWRP7816 isolate of ST101) and the *OXA-181* (two isolates: MUWRP8507 and MUWRP6597 both of ST16). Carbapenemases are members of the molecular class A, B, and D beta-lactamases which can hydrolyze several beta-lactam antibiotics, such as cephalosporins, penicillins, monobactams, and carbapenems. A recent finding of *blaNDM* and the *OXA-181* occurrence has been reported in South Africa and many other places [39]. The *OXA-181* is a member of the *OXA-48* like class D carbapenemases with increasing reports worldwide in the Enterobacteriaceae. Its *OXA-48* group common among *K. pneumoniae* includes, among others, *OXA-48*, *OXA-162*, *OXA-204*, *OXA-232*, *OXA-244*, *OXA-245*, and *OXA-247* that all hydrolyze carbapenems [40]. Increasing reports of these CREs are reported more frequently globally and in Africa presenting challenges for treatment. Previous studies have reported *OXA-48* in Uganda while in other parts of East Africa, carbapenem resistance has been reported to be mainly due to *blaIMP*, *blaVIM-1 blaSPM-l*, *blaNDM-1*, *blaOXA-23*, *blaOXA-24*, *blaOXA-58* and *blaKPC* [13,41]. Similarly, the New Delhi metallo-β-lactamase (*NDM*), β-lactam enzyme able to hydrolyze several available antibiotics was detected in our study. *NDM* was first identified in New Delhi, India, and has since spread globally, causing similar limitations in treatment [42]. Most of these were mobilized on plasmids, as were ESBL genes. Previous studies have shown that *K pneumoniae* carries several multiple-replicon plasmids that have been suggested to provide more ability to carry and disseminate resistance genes and enhance transmission and global dissemination of these pathogens. The fact that all our isolates, except three, carried plasmids signifies a serious problem, especially within healthcare settings, and calls for more dedicated infection control measures to eliminate them from such facilities. Our previous studies have indeed demonstrated a higher prevalence of resistance in *E. coli* from healthcare environments but also from healthcare workers who may be serving as sources of infection with resistant bacteria to hospitalized patients [16].

The threat by MDR *K. pneumoniae* has been recently heightened by their convergence with highly virulent strains. *K. pneumoniae* is thought to have evolved through two different evolutionary pathways, often defined as pathotypes: multidrug-resistant *K. pneumoniae* (MDR-Kp) and hypervirulent *K. pneumoniae* (hvKp) [9]. We found that most of the isolates in this study had several virulence factors including the most common ones. We looked for the genes’ encoding for the siderophores-salmochelin (*iro*), aerobactin (*iuc*), and yersiniabactin (*ybt*)—the iron-foraging molecules—and scored them as defined; we identified two strains with score three, all of which had aerobactin, salmochelin, *rmpADC/rmpA2* and were classified as hypervirulent based on that score. These two strains, MUWRO1041 and MUWRP4500, did not carry resistance to many antibiotics and therefore did not present any hypervirulence and multidrug resistance convergence nuances. Hypervirulence is suspected to be a multifactorial phenotype conferred by the presence of multiple virulence genes carried on large virulence plasmids, some of which simultaneously carry resistance genes [43] and none of which was demonstrated in any of the isolates examined in this study.

## 5. Conclusions

Overall, our study established high genetic diversity among MDR *K. pneuomoniae* isolates collected from patients hospitalized or seeking care at tertiary healthcare facilities in Uganda. Emerging high-risk international pandemic clones (ST11, ST14, ST147, and ST307) were detected in these healthcare settings. We identified very few hypervirulent strains of CG86 and CG412, which fortunately seem not to have converged with the MDR strains, to cause additional management challenges for the infections. Extra efforts for infection prevention and control in most healthcare facilities will be required to minimize their dissemination within the healthcare setting and potential convergence of MDR and hypervirulent strains.

## Data Availability

The nucleotide sequences were submitted to the NCBI database and are available under Bioproject ID: PRJNA1015582.

## Supplementary Materials

The following supporting information can be downloaded at https://www.mdpi.com/article/10.3390/pathogens12111334/s1, Table S1: metadata of the n = 69 isolates considered in this study, Table S2: antibiotic susceptibility of the isolates.
